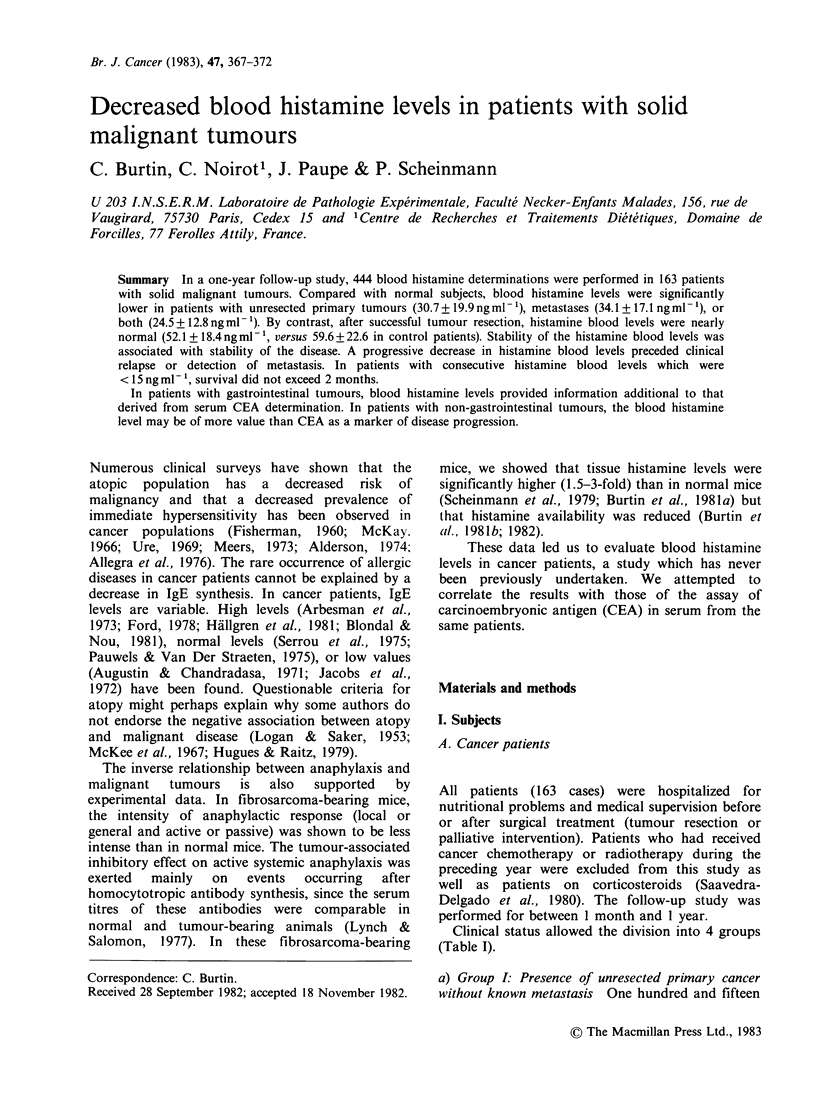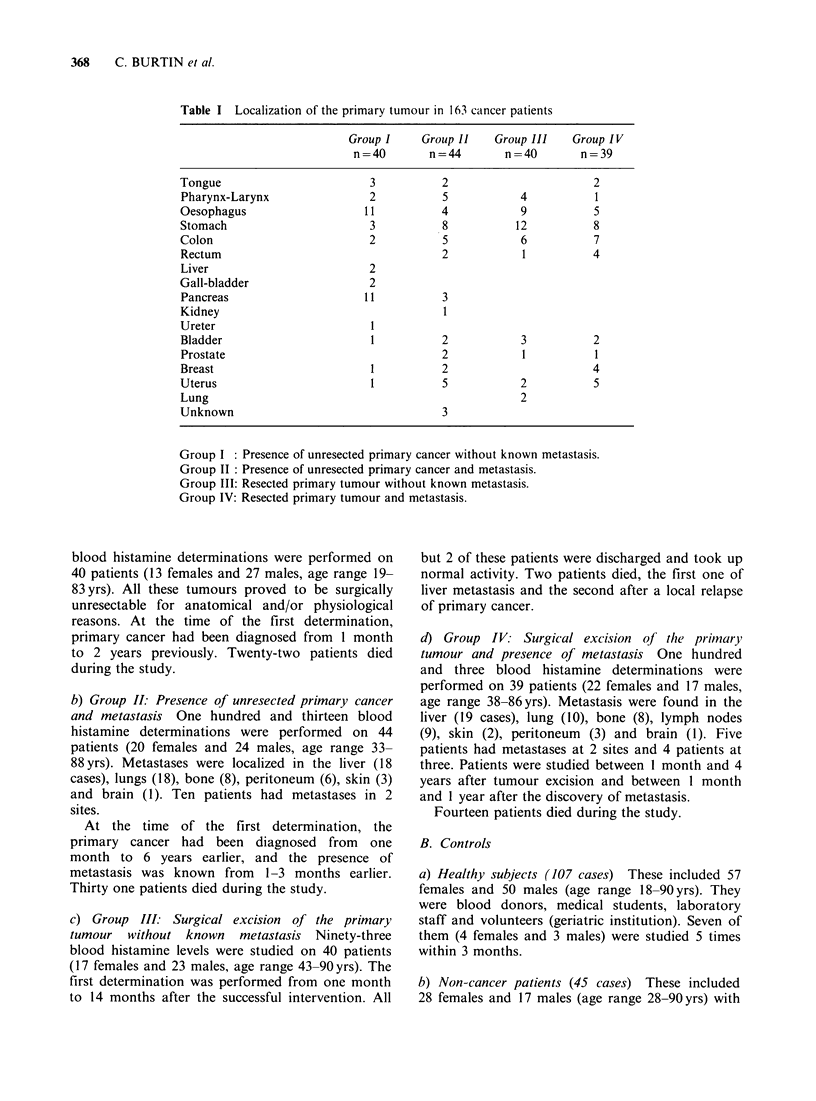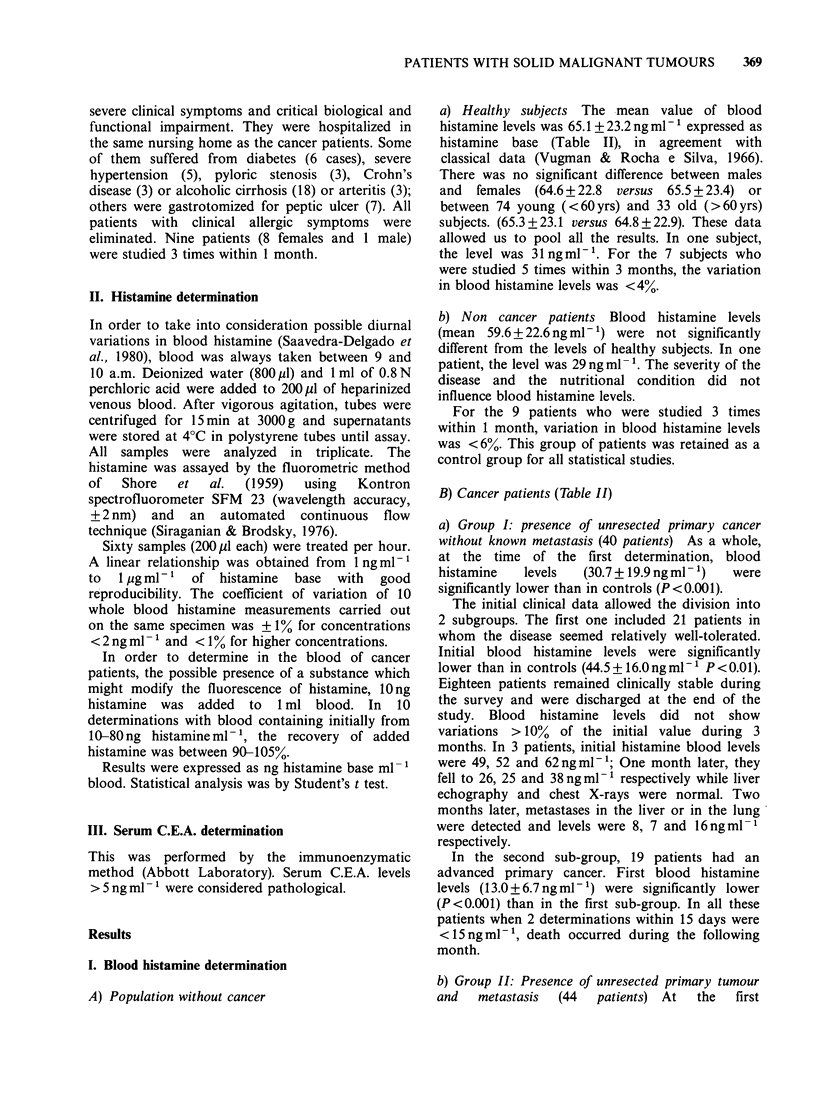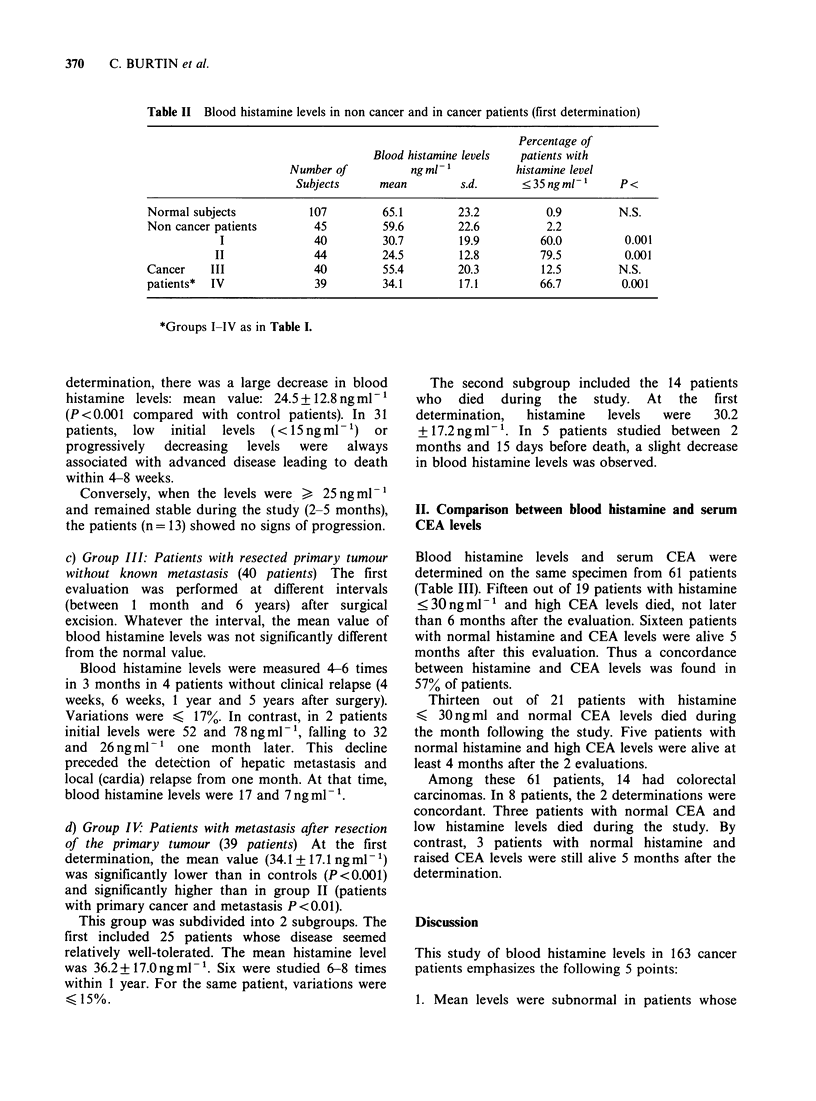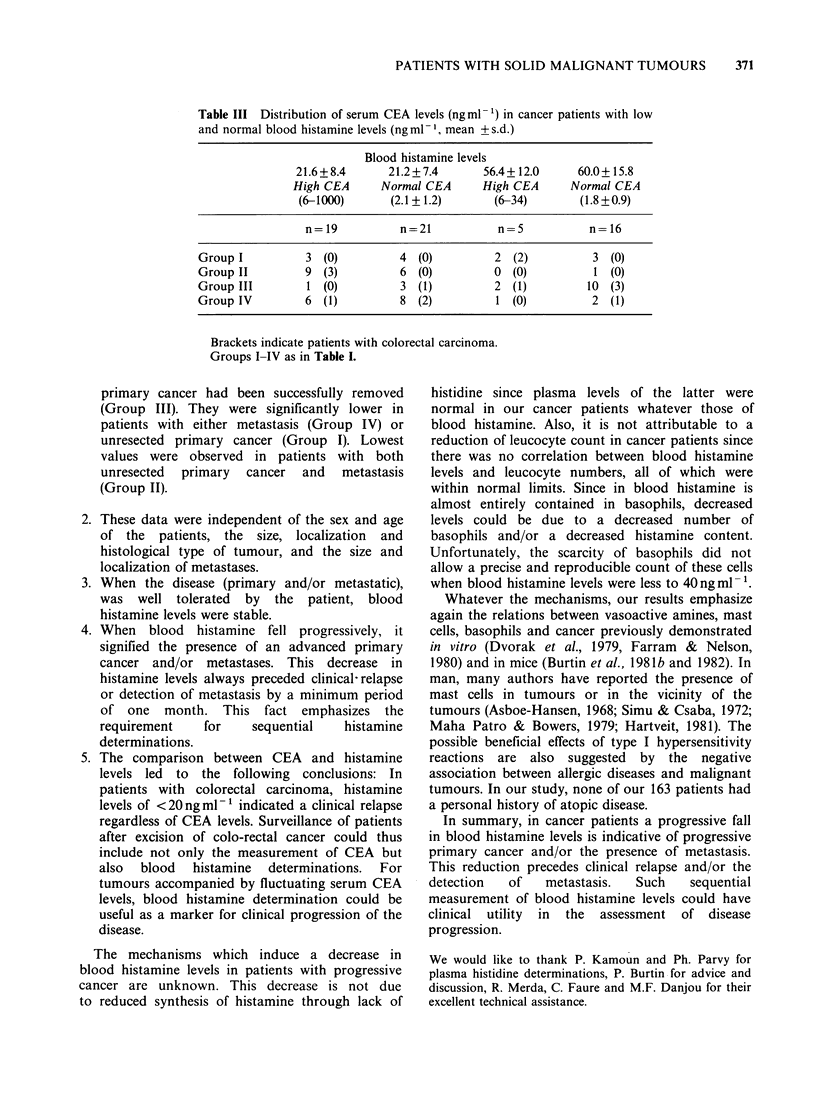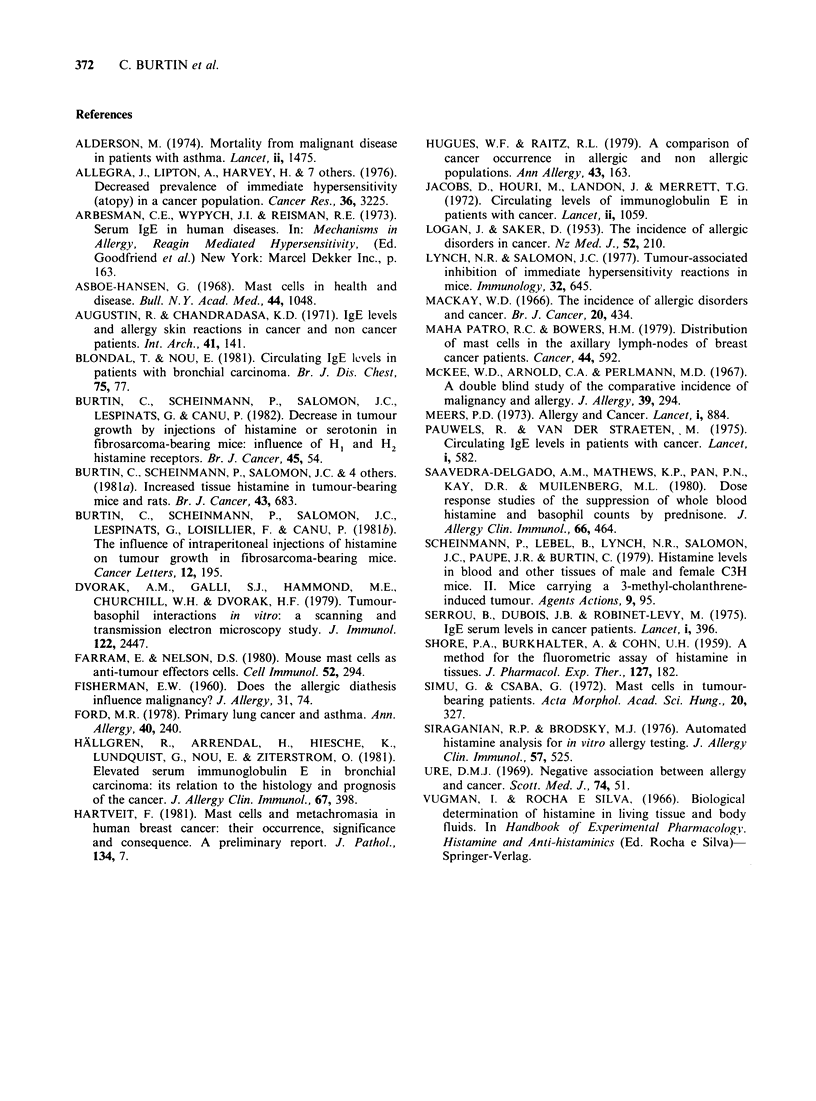# Decreased blood histamine levels in patients with solid malignant tumours.

**DOI:** 10.1038/bjc.1983.55

**Published:** 1983-03

**Authors:** C. Burtin, C. Noirot, J. Paupe, P. Scheinmann

## Abstract

In a one-year follow-up study, 444 blood histamine determinations were performed in 163 patients with solid malignant tumours. Compared with normal subjects, blood histamine levels were significantly lower in patients with unresected primary tumours (30.7 +/- 19.9 ng ml-1), metastases (34.1 +/- 17.1 ng ml-1), or both (24.5 +/- 12.8 ng ml-1). By contrast, after successful tumour resection, histamine blood levels were nearly normal (52.1 +/- 18.4 ng ml-1, versus 59.6 +/- 22.6 in control patients). Stability of the histamine blood levels was associated with stability of the disease. A progressive decrease in histamine blood levels preceded clinical relapse or detection of metastasis. In patients with consecutive histamine blood levels which were less than 15 ng ml-1, survival did not exceed 2 months. In patients with gastrointestinal tumours, blood histamine levels provided information additional to that derived from serum CEA determination. In patients with non-gastrointestinal tumours, the blood histamine level may be of more value than CEA as a marker of disease progression.


*Br. J. Cancer* (1983), **47**, 367–372

### Decreased blood histamine levels in patients with solid malignant tumours

C. Burtin, C. Noirot[1], J. Paupe & P. Scheinmann

*U 203 I.N.S.E.R.M. Laboratoire de Pathologie Expérimentale, Faculté Necker-Enfants Malades, 156, rue de Vaugirard, 75730 Paris, Cedex 15 and [1]Centre de Recherches et Traitements Diététiques, Domaine de Forcilles, 77 Ferolles Attily, France.*

**Summary** In a one-year follow-up study, 444 blood histamine determinations were performed in 163 patients with solid malignant tumours. Compared with normal subjects, blood histamine levels were significantly lower in patients with unresected primary tumours ($30.7 \pm 19.9\,\text{ng ml}^{-1}$), metastases ($34.1 \pm 17.1\,\text{ng ml}^{-1}$), or both ($24.5 \pm 12.8\,\text{ng ml}^{-1}$). By contrast, after successful tumour resection, histamine blood levels were nearly normal ($52.1 \pm 18.4\,\text{ng ml}^{-1}$, *versus* $59.6 \pm 22.6$ in control patients). Stability of the histamine blood levels was associated with stability of the disease. A progressive decrease in histamine blood levels preceded clinical relapse or detection of metastasis. In patients with consecutive histamine blood levels which were $< 15\,\text{ng ml}^{-1}$, survival did not exceed 2 months.

In patients with gastrointestinal tumours, blood histamine levels provided information additional to that derived from serum CEA determination. In patients with non-gastrointestinal tumours, the blood histamine level may be of more value than CEA as a marker of disease progression.

Numerous clinical surveys have shown that the atopic population has a decreased risk of malignancy and that a decreased prevalence of immediate hypersensitivity has been observed in cancer populations (Fisherman, 1960; McKay, 1966; Ure, 1969; Meers, 1973; Alderson, 1974; Allegra *et al.*, 1976). The rare occurrence of allergic diseases in cancer patients cannot be explained by a decrease in IgE synthesis. In cancer patients, IgE levels are variable. High levels (Arbesman *et al.*, 1973; Ford, 1978; Hällgren *et al.*, 1981; Blondal & Nou, 1981), normal levels (Serrou *et al.*, 1975; Pauwels & Van Der Straeten, 1975), or low values (Augustin & Chandradasa, 1971; Jacobs *et al.*, 1972) have been found. Questionable criteria for atopy might perhaps explain why some authors do not endorse the negative association between atopy and malignant disease (Logan & Saker, 1953; McKee *et al.*, 1967; Hugues & Raitz, 1979).

The inverse relationship between anaphylaxis and malignant tumours is also supported by experimental data. In fibrosarcoma-bearing mice, the intensity of anaphylactic response (local or general and active or passive) was shown to be less intense than in normal mice. The tumour-associated inhibitory effect on active systemic anaphylaxis was exerted mainly on events occurring after homocytotropic antibody synthesis, since the serum titres of these antibodies were comparable in normal and tumour-bearing animals (Lynch & Salomon, 1977). In these fibrosarcoma-bearing mice, we showed that tissue histamine levels were significantly higher (1.5–3-fold) than in normal mice (Scheinmann *et al.*, 1979; Burtin *et al.*, 1981*a*) but that histamine availability was reduced (Burtin *et al.*, 1981*b*; 1982).

These data led us to evaluate blood histamine levels in cancer patients, a study which has never been previously undertaken. We attempted to correlate the results with those of the assay of carcinoembryonic antigen (CEA) in serum from the same patients.

### Materials and methods

### I. Subjects

*A. Cancer patients*

All patients (163 cases) were hospitalized for nutritional problems and medical supervision before or after surgical treatment (tumour resection or palliative intervention). Patients who had received cancer chemotherapy or radiotherapy during the preceding year were excluded from this study as well as patients on corticosteroids (Saavedra-Delgado *et al.*, 1980). The follow-up study was performed for between 1 month and 1 year.

Clinical status allowed the division into 4 groups (Table I).

*a) Group I: Presence of unresected primary cancer without known metastasis* One hundred and fifteen

Correspondence: C. Burtin.
Received 28 September 1982; accepted 18 November 1982.

© The Macmillan Press Ltd., 1983

**Table I** Localization of the primary tumour in 163 cancer patients

| | *Group I* n=40 | *Group II* n=44 | *Group III* n=40 | *Group IV* n=39 |
|---|---|---|---|---|
| Tongue | 3 | 2 | | 2 |
| Pharynx-Larynx | 2 | 5 | 4 | 1 |
| Oesophagus | 11 | 4 | 9 | 5 |
| Stomach | 3 | 8 | 12 | 8 |
| Colon | 2 | 5 | 6 | 7 |
| Rectum | | 2 | 1 | 4 |
| Liver | 2 | | | |
| Gall-bladder | 2 | | | |
| Pancreas | 11 | 3 | | |
| Kidney | | 1 | | |
| Ureter | 1 | | | |
| Bladder | 1 | 2 | 3 | 2 |
| Prostate | | 2 | 1 | 1 |
| Breast | 1 | 2 | | 4 |
| Uterus | 1 | 5 | 2 | 5 |
| Lung | | | 2 | |
| Unknown | | 3 | | |

Group I : Presence of unresected primary cancer without known metastasis.
Group II : Presence of unresected primary cancer and metastasis.
Group III: Resected primary tumour without known metastasis.
Group IV: Resected primary tumour and metastasis.

blood histamine determinations were performed on 40 patients (13 females and 27 males, age range 19–83 yrs). All these tumours proved to be surgically unresectable for anatomical and/or physiological reasons. At the time of the first determination, primary cancer had been diagnosed from 1 month to 2 years previously. Twenty-two patients died during the study.

*b) Group II: Presence of unresected primary cancer and metastasis* One hundred and thirteen blood histamine determinations were performed on 44 patients (20 females and 24 males, age range 33–88 yrs). Metastases were localized in the liver (18 cases), lungs (18), bone (8), peritoneum (6), skin (3) and brain (1). Ten patients had metastases in 2 sites.

At the time of the first determination, the primary cancer had been diagnosed from one month to 6 years earlier, and the presence of metastasis was known from 1–3 months earlier. Thirty one patients died during the study.

*c) Group III: Surgical excision of the primary tumour without known metastasis* Ninety-three blood histamine levels were studied on 40 patients (17 females and 23 males, age range 43–90 yrs). The first determination was performed from one month to 14 months after the successful intervention. All but 2 of these patients were discharged and took up normal activity. Two patients died, the first one of liver metastasis and the second after a local relapse of primary cancer.

*d) Group IV: Surgical excision of the primary tumour and presence of metastasis* One hundred and three blood histamine determinations were performed on 39 patients (22 females and 17 males, age range 38–86 yrs). Metastasis were found in the liver (19 cases), lung (10), bone (8), lymph nodes (9), skin (2), peritoneum (3) and brain (1). Five patients had metastases at 2 sites and 4 patients at three. Patients were studied between 1 month and 4 years after tumour excision and between 1 month and 1 year after the discovery of metastasis.

Fourteen patients died during the study.

*B. Controls*

*a) Healthy subjects (107 cases)* These included 57 females and 50 males (age range 18–90 yrs). They were blood donors, medical students, laboratory staff and volunteers (geriatric institution). Seven of them (4 females and 3 males) were studied 5 times within 3 months.

*b) Non-cancer patients (45 cases)* These included 28 females and 17 males (age range 28–90 yrs) with severe clinical symptoms and critical biological and functional impairment. They were hospitalized in the same nursing home as the cancer patients. Some of them suffered from diabetes (6 cases), severe hypertension (5), pyloric stenosis (3), Crohn's disease (3) or alcoholic cirrhosis (18) or arteritis (3); others were gastrotomized for peptic ulcer (7). All patients with clinical allergic symptoms were eliminated. Nine patients (8 females and 1 male) were studied 3 times within 1 month.

### II. Histamine determination

In order to take into consideration possible diurnal variations in blood histamine (Saavedra-Delgado *et al.*, 1980), blood was always taken between 9 and 10 a.m. Deionized water (800 $\mu$l) and 1 ml of 0.8 N perchloric acid were added to 200 $\mu$l of heparinized venous blood. After vigorous agitation, tubes were centrifuged for 15 min at 3000 g and supernatants were stored at 4°C in polystyrene tubes until assay. All samples were analyzed in triplicate. The histamine was assayed by the fluorometric method of Shore *et al.* (1959) using Kontron spectrofluorometer SFM 23 (wavelength accuracy, $\pm$2 nm) and an automated continuous flow technique (Siraganian & Brodsky, 1976).

Sixty samples (200 $\mu$l each) were treated per hour. A linear relationship was obtained from 1 ng ml$^{-1}$ to 1 $\mu$g ml$^{-1}$ of histamine base with good reproducibility. The coefficient of variation of 10 whole blood histamine measurements carried out on the same specimen was $\pm$1% for concentrations $<$2 ng ml$^{-1}$ and $<$1% for higher concentrations.

In order to determine in the blood of cancer patients, the possible presence of a substance which might modify the fluorescence of histamine, 10 ng histamine was added to 1 ml blood. In 10 determinations with blood containing initially from 10–80 ng histamine ml$^{-1}$, the recovery of added histamine was between 90–105%.

Results were expressed as ng histamine base ml$^{-1}$ blood. Statistical analysis was by Student's *t* test.

### III. Serum C.E.A. determination

This was performed by the immunoenzymatic method (Abbott Laboratory). Serum C.E.A. levels $>$5 ng ml$^{-1}$ were considered pathological.

### Results

### I. Blood histamine determination

*A) Population without cancer*

*a) Healthy subjects* The mean value of blood histamine levels was 65.1$\pm$23.2 ng ml$^{-1}$ expressed as histamine base (Table II), in agreement with classical data (Vugman & Rocha e Silva, 1966). There was no significant difference between males and females (64.6$\pm$22.8 *versus* 65.5$\pm$23.4) or between 74 young ($<$60 yrs) and 33 old ($>$60 yrs) subjects. (65.3$\pm$23.1 *versus* 64.8$\pm$22.9). These data allowed us to pool all the results. In one subject, the level was 31 ng ml$^{-1}$. For the 7 subjects who were studied 5 times within 3 months, the variation in blood histamine levels was $<$4%.

*b) Non cancer patients* Blood histamine levels (mean 59.6$\pm$22.6 ng ml$^{-1}$) were not significantly different from the levels of healthy subjects. In one patient, the level was 29 ng ml$^{-1}$. The severity of the disease and the nutritional condition did not influence blood histamine levels.

For the 9 patients who were studied 3 times within 1 month, variation in blood histamine levels was $<$6%. This group of patients was retained as a control group for all statistical studies.

*B) Cancer patients (Table II)*

*a) Group I: presence of unresected primary cancer without known metastasis (40 patients)* As a whole, at the time of the first determination, blood histamine levels (30.7$\pm$19.9 ng ml$^{-1}$) were significantly lower than in controls ($P<0.001$).

The initial clinical data allowed the division into 2 subgroups. The first one included 21 patients in whom the disease seemed relatively well-tolerated. Initial blood histamine levels were significantly lower than in controls (44.5$\pm$16.0 ng ml$^{-1}$ $P<0.01$). Eighteen patients remained clinically stable during the survey and were discharged at the end of the study. Blood histamine levels did not show variations $>$10% of the initial value during 3 months. In 3 patients, initial histamine blood levels were 49, 52 and 62 ng ml$^{-1}$; One month later, they fell to 26, 25 and 38 ng ml$^{-1}$ respectively while liver echography and chest X-rays were normal. Two months later, metastases in the liver or in the lung were detected and levels were 8, 7 and 16 ng ml$^{-1}$ respectively.

In the second sub-group, 19 patients had an advanced primary cancer. First blood histamine levels (13.0$\pm$6.7 ng ml$^{-1}$) were significantly lower ($P<0.001$) than in the first sub-group. In all these patients when 2 determinations within 15 days were $<$15 ng ml$^{-1}$, death occurred during the following month.

*b) Group II: Presence of unresected primary tumour and metastasis (44 patients)* At the first

**Table II** Blood histamine levels in non cancer and in cancer patients (first determination)

| | | Number of Subjects | Blood histamine levels $ng\,ml^{-1}$ mean | s.d. | Percentage of patients with histamine level $\leq 35\,ng\,ml^{-1}$ | $P<$ |
|---|---|---|---|---|---|---|
| Normal subjects | | 107 | 65.1 | 23.2 | 0.9 | N.S. |
| Non cancer patients | | 45 | 59.6 | 22.6 | 2.2 | |
| | I | 40 | 30.7 | 19.9 | 60.0 | 0.001 |
| | II | 44 | 24.5 | 12.8 | 79.5 | 0.001 |
| Cancer | III | 40 | 55.4 | 20.3 | 12.5 | N.S. |
| patients* | IV | 39 | 34.1 | 17.1 | 66.7 | 0.001 |

*Groups I–IV as in **Table I.**

determination, there was a large decrease in blood histamine levels: mean value: $24.5 \pm 12.8\,ng\,ml^{-1}$ ($P<0.001$ compared with control patients). In 31 patients, low initial levels ($<15\,ng\,ml^{-1}$) or progressively decreasing levels were always associated with advanced disease leading to death within 4–8 weeks.

Conversely, when the levels were $\geqslant 25\,ng\,ml^{-1}$ and remained stable during the study (2–5 months), the patients (n = 13) showed no signs of progression.

*c) Group III: Patients with resected primary tumour without known metastasis (40 patients)* The first evaluation was performed at different intervals (between 1 month and 6 years) after surgical excision. Whatever the interval, the mean value of blood histamine levels was not significantly different from the normal value.

Blood histamine levels were measured 4–6 times in 3 months in 4 patients without clinical relapse (4 weeks, 6 weeks, 1 year and 5 years after surgery). Variations were $\leqslant$ 17%. In contrast, in 2 patients initial levels were 52 and $78\,ng\,ml^{-1}$, falling to 32 and $26\,ng\,ml^{-1}$ one month later. This decline preceded the detection of hepatic metastasis and local (cardia) relapse from one month. At that time, blood histamine levels were 17 and $7\,ng\,ml^{-1}$.

*d) Group IV: Patients with metastasis after resection of the primary tumour (39 patients)* At the first determination, the mean value ($34.1 \pm 17.1\,ng\,ml^{-1}$) was significantly lower than in controls ($P<0.001$) and significantly higher than in group II (patients with primary cancer and metastasis $P<0.01$).

This group was subdivided into 2 subgroups. The first included 25 patients whose disease seemed relatively well-tolerated. The mean histamine level was $36.2 \pm 17.0\,ng\,ml^{-1}$. Six were studied 6–8 times within 1 year. For the same patient, variations were $\leqslant$15%.

The second subgroup included the 14 patients who died during the study. At the first determination, histamine levels were 30.2 $\pm 17.2\,ng\,ml^{-1}$. In 5 patients studied between 2 months and 15 days before death, a slight decrease in blood histamine levels was observed.

### II. Comparison between blood histamine and serum CEA levels

Blood histamine levels and serum CEA were determined on the same specimen from 61 patients (Table III). Fifteen out of 19 patients with histamine $\leq 30\,ng\,ml^{-1}$ and high CEA levels died, not later than 6 months after the evaluation. Sixteen patients with normal histamine and CEA levels were alive 5 months after this evaluation. Thus a concordance between histamine and CEA levels was found in 57% of patients.

Thirteen out of 21 patients with histamine $\leqslant$ 30 ng ml and normal CEA levels died during the month following the study. Five patients with normal histamine and high CEA levels were alive at least 4 months after the 2 evaluations.

Among these 61 patients, 14 had colorectal carcinomas. In 8 patients, the 2 determinations were concordant. Three patients with normal CEA and low histamine levels died during the study. By contrast, 3 patients with normal histamine and raised CEA levels were still alive 5 months after the determination.

### Discussion

This study of blood histamine levels in 163 cancer patients emphasizes the following 5 points:

1. Mean levels were subnormal in patients whose

**Table III** Distribution of serum CEA levels (ng $ml^{-1}$) in cancer patients with low and normal blood histamine levels (ng $ml^{-1}$, mean ±s.d.)

| | Blood histamine levels | | | |
|---|---|---|---|---|
| | 21.6±8.4 *High CEA* (6–1000) | 21.2±7.4 *Normal CEA* (2.1±1.2) | 56.4±12.0 *High CEA* (6–34) | 60.0±15.8 *Normal CEA* (1.8±0.9) |
| | n=19 | n=21 | n=5 | n=16 |
| Group I | 3 (0) | 4 (0) | 2 (2) | 3 (0) |
| Group II | 9 (3) | 6 (0) | 0 (0) | 1 (0) |
| Group III | 1 (0) | 3 (1) | 2 (1) | 10 (3) |
| Group IV | 6 (1) | 8 (2) | 1 (0) | 2 (1) |

Brackets indicate patients with colorectal carcinoma.
Groups I–IV as in **Table I.**

primary cancer had been successfully removed (Group III). They were significantly lower in patients with either metastasis (Group IV) or unresected primary cancer (Group I). Lowest values were observed in patients with both unresected primary cancer and metastasis (Group II).

2. These data were independent of the sex and age of the patients, the size, localization and histological type of tumour, and the size and localization of metastases.
3. When the disease (primary and/or metastatic), was well tolerated by the patient, blood histamine levels were stable.
4. When blood histamine fell progressively, it signified the presence of an advanced primary cancer and/or metastases. This decrease in histamine levels always preceded clinical relapse or detection of metastasis by a minimum period of one month. This fact emphasizes the requirement for sequential histamine determinations.
5. The comparison between CEA and histamine levels led to the following conclusions: In patients with colorectal carcinoma, histamine levels of $<20$ ng $ml^{-1}$ indicated a clinical relapse regardless of CEA levels. Surveillance of patients after excision of colo-rectal cancer could thus include not only the measurement of CEA but also blood histamine determinations. For tumours accompanied by fluctuating serum CEA levels, blood histamine determination could be useful as a marker for clinical progression of the disease.

The mechanisms which induce a decrease in blood histamine levels in patients with progressive cancer are unknown. This decrease is not due to reduced synthesis of histamine through lack of histidine since plasma levels of the latter were normal in our cancer patients whatever those of blood histamine. Also, it is not attributable to a reduction of leucocyte count in cancer patients since there was no correlation between blood histamine levels and leucocyte numbers, all of which were within normal limits. Since in blood histamine is almost entirely contained in basophils, decreased levels could be due to a decreased number of basophils and/or a decreased histamine content. Unfortunately, the scarcity of basophils did not allow a precise and reproducible count of these cells when blood histamine levels were less to 40 ng $ml^{-1}$.

Whatever the mechanisms, our results emphasize again the relations between vasoactive amines, mast cells, basophils and cancer previously demonstrated *in vitro* (Dvorak *et al.*, 1979, Farram & Nelson, 1980) and in mice (Burtin *et al.*, 1981*b* and 1982). In man, many authors have reported the presence of mast cells in tumours or in the vicinity of the tumours (Asboe-Hansen, 1968; Simu & Csaba, 1972; Maha Patro & Bowers, 1979; Hartveit, 1981). The possible beneficial effects of type I hypersensitivity reactions are also suggested by the negative association between allergic diseases and malignant tumours. In our study, none of our 163 patients had a personal history of atopic disease.

In summary, in cancer patients a progressive fall in blood histamine levels is indicative of progressive primary cancer and/or the presence of metastasis. This reduction precedes clinical relapse and/or the detection of metastasis. Such sequential measurement of blood histamine levels could have clinical utility in the assessment of disease progression.

We would like to thank P. Kamoun and Ph. Parvy for plasma histidine determinations, P. Burtin for advice and discussion, R. Merda, C. Faure and M.F. Danjou for their excellent technical assistance.

### References

ALDERSON, M. (1974). Mortality from malignant disease in patients with asthma. *Lancet*, **ii**, 1475.

ALLEGRA, J., LIPTON, A., HARVEY, H. & 7 others. (1976). Decreased prevalence of immediate hypersensitivity (atopy) in a cancer population. *Cancer Res.*, **36**, 3225.

ARBESMAN, C.E., WYPYCH, J.I. & REISMAN, R.E. (1973). Serum IgE in human diseases. In: *Mechanisms in Allergy, Reagin Mediated Hypersensitivity*, (Ed. Goodfriend *et al.*) New York: Marcel Dekker Inc., p. 163.

ASBOE-HANSEN, G. (1968). Mast cells in health and disease. *Bull. N.Y. Acad. Med.*, **44**, 1048.

AUGUSTIN, R. & CHANDRADASA, K.D. (1971). IgE levels and allergy skin reactions in cancer and non cancer patients. *Int. Arch.*, **41**, 141.

BLONDAL, T. & NOU, E. (1981). Circulating IgE levels in patients with bronchial carcinoma. *Br. J. Dis. Chest*, **75**, 77.

BURTIN, C., SCHEINMANN, P., SALOMON, J.C., LESPINATS, G. & CANU, P. (1982). Decrease in tumour growth by injections of histamine or serotonin in fibrosarcoma-bearing mice: influence of $H_1$ and $H_2$ histamine receptors. *Br. J. Cancer*, **45**, 54.

BURTIN, C., SCHEINMANN, P., SALOMON, J.C. & 4 others. (1981*a*). Increased tissue histamine in tumour-bearing mice and rats. *Br. J. Cancer*, **43**, 683.

BURTIN, C., SCHEINMANN, P., SALOMON, J.C., LESPINATS, G., LOISILLIER, F. & CANU, P. (1981*b*). The influence of intraperitoneal injections of histamine on tumour growth in fibrosarcoma-bearing mice. *Cancer Letters*, **12**, 195.

DVORAK, A.M., GALLI, S.J., HAMMOND, M.E., CHURCHILL, W.H. & DVORAK, H.F. (1979). Tumour-basophil interactions *in vitro*: a scanning and transmission electron microscopy study. *J. Immunol.* **122**, 2447.

FARRAM, E. & NELSON, D.S. (1980). Mouse mast cells as anti-tumour effectors cells. *Cell Immunol.* **52**, 294.

FISHERMAN, E.W. (1960). Does the allergic diathesis influence malignancy? *J. Allergy*, 31, 74.

FORD, M.R. (1978). Primary lung cancer and asthma. *Ann. Allergy*, **40**, 240.

HÄLLGREN, R., ARRENDAL, H., HIESCHE, K., LUNDQUIST, G., NOU, E. & ZITERSTROM, O. (1981). Elevated serum immunoglobulin E in bronchial carcinoma: its relation to the histology and prognosis of the cancer. *J. Allergy Clin. Immunol.*, **67**, 398.

HARTVEIT, F. (1981). Mast cells and metachromasia in human breast cancer: their occurrence, significance and consequence. A preliminary report. *J. Pathol.*, **134**, 7.

HUGUES, W.F. & RAITZ, R.L. (1979). A comparison of cancer occurrence in allergic and non allergic populations. *Ann Allergy*, **43**, 163.

JACOBS, D., HOURI, M., LANDON, J. & MERRETT, T.G. (1972). Circulating levels of immunoglobulin E in patients with cancer. *Lancet*, **ii**, 1059.

LOGAN, J. & SAKER, D. (1953). The incidence of allergic disorders in cancer. *Nz Med. J.*, **52**, 210.

LYNCH, N.R. & SALOMON, J.C. (1977). Tumour-associated inhibition of immediate hypersensitivity reactions in mice. *Immunology*, **32**, 645.

MACKAY, W.D. (1966). The incidence of allergic disorders and cancer. *Br. J. Cancer*, **20**, 434.

MAHA PATRO, R.C. & BOWERS, H.M. (1979). Distribution of mast cells in the axillary lymph-nodes of breast cancer patients. *Cancer*, **44**, 592.

McKEE, W.D., ARNOLD, C.A. & PERLMANN, M.D. (1967). A double blind study of the comparative incidence of malignancy and allergy. *J. Allergy*, **39**, 294.

MEERS, P.D. (1973). Allergy and Cancer. *Lancet*, **i**, 884.

PAUWELS, R. & VAN DER STRAETEN, M. (1975). Circulating IgE levels in patients with cancer. *Lancet*, **i**, 582.

SAAVEDRA-DELGADO, A.M., MATHEWS, K.P., PAN, P.N., KAY, D.R. & MUILENBERG, M.L. (1980). Dose response studies of the suppression of whole blood histamine and basophil counts by prednisone. *J. Allergy Clin. Immunol.*, **66**, 464.

SCHEINMANN, P., LEBEL, B., LYNCH, N.R., SALOMON, J.C., PAUPE, J.R. & BURTIN, C. (1979). Histamine levels in blood and other tissues of male and female C3H mice. II. Mice carrying a 3-methyl-cholanthrene-induced tumour. *Agents Actions*, **9**, 95.

SERROU, B., DUBOIS, J.B. & ROBINET-LEVY, M. (1975). IgE serum levels in cancer patients. *Lancet*, **i**, 396.

SHORE, P.A., BURKHALTER, A. & COHN, U.H. (1959). A method for the fluorometric assay of histamine in tissues. *J. Pharmacol. Exp. Ther.*, **127**, 182.

SIMU, G. & CSABA, G. (1972). Mast cells in tumour-bearing patients. *Acta Morphol. Acad. Sci. Hung.*, **20**, 327.

SIRAGANIAN, R.P. & BRODSKY, M.J. (1976). Automated histamine analysis for *in vitro* allergy testing. *J. Allergy Clin. Immunol.*, **57**, 525.

URE, D.M.J. (1969). Negative association between allergy and cancer. *Scott. Med. J.*, **74**, 51.

VUGMAN, I. & ROCHA E SILVA, (1966). Biological determination of histamine in living tissue and body fluids. In *Handbook of Experimental Pharmacology. Histamine and Anti-histaminics* (Ed. Rocha e Silva)—Springer-Verlag.